# Moving for optimal immunity: the effect of acute high-intensity interval training on phenotype, virus specificity and chemokine receptor expression in human CD8+ T cells

**DOI:** 10.3389/fimmu.2025.1739657

**Published:** 2026-01-19

**Authors:** Katharina Leuchte, Thy Viet Luu, Sara Fresnillo Saló, Kasper Madsen, Lise Heide-Ottosen, Signe Koggersbøl Skadborg, Janine Sophie Kemming, Morten Orebo Holmström, Hongjin Chen, Lars Rønn Olsen, Anders Vinther, Mads Hald Andersen, Sine Reker Hadrup, Per thor Straten, Gitte Holmen Olofsson

**Affiliations:** 1 National Center for Cancer Immune Therapy (CCIT-DK), Department of Oncology, Copenhagen University Hospital Herlev, Herlev, Denmark; 2 Department I of Internal Medicine, Medical Faculty and University Hospital of Cologne, University of Cologne, Cologne, Germany; 3 Department of Oncology, Copenhagen University Hospital Herlev, Herlev, Denmark; 4 Department of Health Technology, Section of Experimental and Translational Immunology, Technical University of Denmark, Kongens Lyngby, Denmark; 5 Department of Health Technology, Bioinformatics, Single Cell Omics, Technical University of Denmark, Kongens Lyngby, Denmark; 6 Department of Immunology and Microbiology, LEO Foundation Skin Immunology Research Center, University of Copenhagen, Copenhagen, Denmark; 7 Department of Physiotherapy and Occupational Therapy, Copenhagen University Hospitals Herlev and Gentofte, Herlev, Denmark; 8 Department of Clinical Medicine, University of Copenhagen, Copenhagen, Denmark

**Keywords:** acute exercise, adaptive immunity, chemokine, endurance exercise, exercise immunology, physical activity

## Abstract

**Introduction:**

Physical activity induces rapid and selective leukocyte mobilization. Among the most responsive cell types to high-intensity exercise are CD8^+^ T cells, key effectors of immune defense against infected cells and cancer. However, comprehensive profiling of acute high-intensity interval training (HIIT)-induced modulation of the CD8^+^ T cell compartment remains lacking.

**Methods:**

We assessed the effects of a supervised, group-based HIIT session on the CD8+ T cell compartment in 23 healthy participants. Blood was collected at baseline, immediately post-exercise (ex02), and one hour post-exercise (ex60). CD8^+^ T cells were analyzed for virus peptide reactivity using DNA-barcoded peptide-MHC multimer staining targeting 250 peptides. Differentiation status, chemokine receptor expression, and ligand regulation were assessed by flow cytometry and Olink proteomics, and finally, associations between individual characteristics and CD8^+^ T cell mobilization were analyzed.

**Results:**

A single HIIT bout induced robust CD8^+^ T cell mobilization followed by substantial egress, which were consistent across fitness levels, body composition and age. Circulating virus-reactive T cells significantly increased in peripheral blood in response to exercise across virus types, including EBV-, SARS-CoV-2- and CMV-specific T cells. HIIT modulated chemokine receptor profiles, and memory subsets were reorganized, reducing terminally differentiated and CD57^+^, PD-1^+^, and CD28^neg^ cells at ex60 post-exercise. Notably, catecholamines NE and EPI peaked post-exercise, and NE was selectively associated with CD8^+^ T cell mobilization.

**Discussion:**

In conclusion, acute HIIT mobilizes functional, virus-reactive CD8^+^ T cells with features indicative of enhanced migratory and activation potential, supporting translational use from tumor immunology to infectious disease. The study is registered at clinicaltrials.gov (NCT05826496).

## 1 Introduction

While the positive effects of physical activity in humans are well studied (1), thereby forming an important pillar of disease prevention, the mechanism of exercise-induced immune regulation is considerably less studied. Physically active individuals have augmented immune competence such as improved response to vaccination (2, 3) and HIV therapy (4), lower chronic inflammation (5, 6), and reduced cancer incidence (7), reflecting the profound interplay between exercise and optimal immunity.

The human immune system is an intricate and dynamic network, capable of rapid adaptation to external stimuli. As such, endurance exercise at high intensity effectively mobilizes leukocytes into the peripheral blood in an intensity-dependent and cell type-selective manner (8). Mobilization also occurs according to differentiation status, with later-differentiated CD8+ T cells being preferentially mobilized (9, 10). Since these compartments contain most virus-specific effector cells, this is functionally relevant for antiviral surveillance (9, 11). This acute mobilization phase is typically followed by a nadir (12), reflecting the redistribution of leukocytes to peripheral tissues, including sites of inflammation, repair, or tumors (13, 14). CD8+ T cells – mediators of adaptive immunity (15–17) – are among the most responsive immune cell types to acute high-intensity interval training (HIIT) (8).

While catecholamines are known to drive leukocyte mobilization via adrenergic signaling (10), CD8+ T cells’ cytotoxic function also depends on their capacity to migrate from peripheral blood into tissues, a process critically orchestrated by chemokine receptor–ligand interactions (18). Receptors, including CCR5 and CX3CR1, guide this trafficking, a response that might vary with exercise frequency, intensity, time, and type (FITT) (19). Characterizing the distinct chemokine receptor profile of exercise-mobilized CD8+ T cells and abundance of their ligands is thus pivotal to understanding their capacity for tissue infiltration and role in local immune surveillance.

Exercise-induced changes in the immune system have many potential applications: These include optimizing vaccination strategies, improving adoptive cell therapy for hematological cancers (20) and responses to cancer immunotherapy (21), preventing infectious diseases and modulating autoimmune conditions. Additionally, they may help manage chronic inflammation-related diseases, such as type 2 diabetes and cardiovascular diseases, promote wound healing and slow down immune, organ and organism ageing (1, 22–24).

Our study has three aims: (1) to systematically profile the exercise-induced mobilization of CD8+ T cells, (2) to investigate the exercise-mobilized CD8+ T cells’ phenotype and virus specificity, (3) to identify the exercise-induced dynamics in chemokine receptor expression and their ligands, overall in association with individual factors. Ultimately our goal is to advance our understanding of personalized CD8+ T cell responses to acute high-intensity endurance training.

## 2 Materials and methods

### 2.1 Study participants and preparation procedures

**Figure 1:**
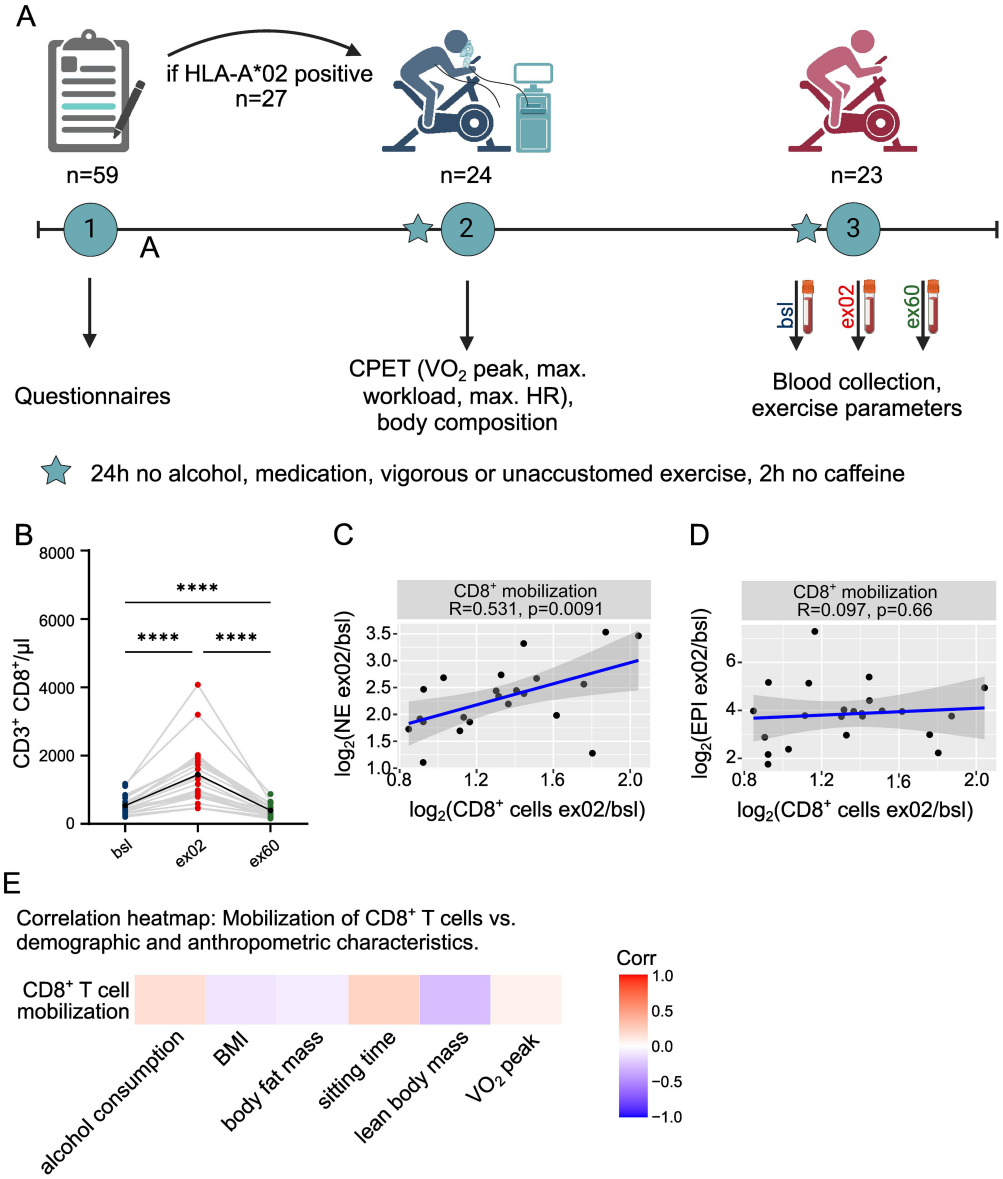
Acute HIIT-induced CD8^+^ T cell mobilization is selectively associated with norepinephrine peak, and independent of fitness and anthropometrics. **(A)** INHALE study design. **(B)** Acute HIIT-induced CD8^+^ T cell dynamics. **(C, D)** Scatterplots showing the relationship between CD8^+^ T cell and serum catecholamine increase. R indicates Pearson’s correlation coefficient, p statistical significance. Plotted is log2(ex02/bsl). **(E)** Age-adjusted correlation heatmap of CD8^+^ T cell mobilization vs. demographic, anthropometric and fitness participant characteristics. Characteristics were assessed at inclusion or at time of CPET. n=23; Significance levels indicated by asterisks on the graphs: *p ≤ 0.05, **p ≤ 0.01, ***p ≤ 0.001, ****p ≤ 0.0001; only statistically significant differences are indicated. CPET cardiopulmonary exercise test, EPI epinephrine, HIIT High-intensity interval training, NE norepinephrine, VO2peak peak oxygen consumption.

A total of 59 healthy individuals (age 21–68) were included in the INHALE study between March and April 2023. Participants were eligible if they were between 18 and 75 years old, able to understand English or Danish, free of exercise-limiting or unstable medical conditions, free of autoimmune diseases requiring active treatment and excluded if using medications affecting cardiovascular, metabolic, or immune responses (e.g., >10 mg prednisone equivalents, immunosuppressive medications or beta blockers). Written informed consent was obtained. Health status and medication, and demographic, lifestyle, and habitual physical activity data were collected using the International Physical Activity Questionnaire (IPAQ). Participants were then selected to be HLA-A2 positive to maximize detection of virus-reactive CD8^+^ T cells restricted by the applied pMHC multimer panel (see below). HLA-A2 status was determined by flow cytometry analysis (see below) of capillary EDTA blood (Sarstedt); 27 (46.6%) were positive, of whom 23 (10 females, 13 males) aged 25–65 continued (**Table 1**). Study design is shown in **Figure 1A**. The study is registered at clinicaltrials.gov (NCT05826496) with ethics approval from Capital Region’s Ethics Board (H-23006672) and Danish Data Protection Agency (P-2023-95).

**Table 1:** Participant characteristics in the INHALE study.

Parameters	Number
Demographic characteristics	
Age – years	
Mean	37.3
Range	25-65
Sex - no. (%)	
Male	13 (56.5)
Female	10 (43.5)
Country of birth	
Denmark	15
Other	8
Civil status	
Married/in a relationship	19 (82.6)
Single/divorced/widowed	4 (17.4)
Highest level of education	
Trade/Technical/Vocational education	1 (4.3)
Short-term higher education (less than 3 years)	1 (4.3)
Medium higher education (3–4 years)	8 (34.8)
Long-term higher education (5 years or more)	13 (56.5)
Employment status	
Employed/self-employed	20 (87)
Unemployed/homemaker/student	2 (8.7)
Retired	1 (4.3)
Behavioral characteristics	
Smoking status - no. (%)	
Never-smoker	10 (43.5)
Former smoker (stopped >=4 weeks)	7 (30.4)
Active daily smoker	0
Active “only on special occasions” smoker	6 (26.1)
Alcohol consumption - items/week	
Mean	6.4
Range	0-20
Exercise history	
Level of physical activity - MET-min/week	
Mean	3585
Range	240-13320
Sedentary time - h/weekday	
Mean	8
Range	2-12
Anthropometric characteristics	
BMI - kg/m^2^	
Mean	23.4
Range	19.3-27.3
18.5 - 24.9	17 (73.9)
25.0-29.9	6 (26.1)
>30	0
Fat percentage - %	
Mean	24.4
Range	12.7 - 34.2
Fat mass – kg	
Mean	18.1
Range	9.6 - 25.9

BMI body mass index, MET metabolic equivalent.

### 2.2 Cardiopulmonary exercise test

HLA-A*02 positive participants completed a CPET on a cycling ergometer (CPET Vyntus CPX, Ergometer ViaSprint 150p, Vyarie Medical) following 24 hours of avoiding alcohol, non-prescription meds, vigorous/unaccustomed exercise, and 2 hours caffeine fasting. Based on estimated aerobic capacity appropriate incremental workload protocols were chosen aiming for a test duration of 8–12 minutes and terminated at volitional exhaustion (25). Ventilation, heart rate (HR), and respiratory exchange rate (RER) were recorded every 15 s; VO2 peak, maximal power output, and HR were calculated as the average of a 30s period based on 2 subsequent measurements (25) and confirmed visually by three independent researchers. Anthropometric and body composition measurements by bioimpedance (SoZo, ImpediaMedia) were performed at CPET.

### 2.3 Exercise session and blood sampling

Within 24 days post-CPET, participants completed a physiotherapist-supervised, group-based exercise session after similar abstinence and fasting conditions, which took place during daylight between 8:00 and 14:00 to minimize bias by circadian rhythm. Baseline blood samples were collected by venipuncture at arrival. The warm-up included 5 minutes on a bicycle ergometer followed by 5 × 2-minute circuit training on: 1) air bike ergometer, 2) ski ergometer, 3) rowing ergometer, 4) cross trainer, and 5) step bench exercises. All warm-up exercises were self-paced but overseen by an experienced physiotherapist encouraging the participants to exercise with sufficient intensity to substantially increase HR and respiration. Bicycle training (Motion Cross 600, Emotion Fitness) started with 2 minutes low workload pedaling (approximately 50% of the maximal power output at CPET), then 3 × 3-minute high-intensity intervals alternating 20 s “all-out” (aiming at 100% of the maximal power output at CPET) with 20 s easy pedaling (aiming at 50%). These intervals were separated by two 3-minute steady-state sequences at 50-70% of the maximal power output during CPET. Resistance and cadence were pre-set to meet or exceed target workloads. Blood samples were taken at baseline (bsl), within 2 minutes post-exercise (ex02), and after 60 minutes (ex60) (Vacutainer Sodium Heparin, BD; Vacuette Serum, Greiner bio-one). Only water was consumed until ex60.

### 2.4 Blood processing

Samples were processed immediately at the sterile laboratory. Serum was obtained by centrifugation at 1300 g for 10 min, aliquoted within 1 h, and stored at –80°C. PBMCs were isolated using density gradient centrifugation in Leukosep tubes (Greiner bio-one) and counted. PBMCs were then cryopreserved in inactivated human AB serum (Sigma Aldrich) with 10% DMSO (Honeywell), and stored at –150°C. HLA genotyping was performed by DKMS Life Science Lab (Dresden, Germany) using amplicon sequencing.

### 2.5 Flow cytometry

For HLA-A2 staining, in short, 50µl whole blood were incubated with 2µl antibody (20 minutes, dark, room temperature), afterwards 2ml BD lysis buffer (BD Biosciences) were added. After 10 minutes, cells were resuspended in FACS buffer and acquired on the NovoCyte Quanteon (Agilent).Whole blood was stained with a 6-color TBNK panel in Trucount tubes (**Supplementary Table S1**) according to the manufacturer’s protocol and acquired on a BD FACSCanto II. Gating was standardized (FACSCanto Software, BD). Expression of CCR2, CCR5, CXCL2, CXCL4, CXCL6 and CX3CR1 was analyzed in cryopreserved PBMCs (antibodies listed in **Supplementary Table S1**). Cells were washed twice with PBS/2% FCS (FACS buffer), stained with chemokine receptor antibodies at 37°C for 15 min, then with antibodies targeting CD3, CD4, CD8, CD28 and CCR7 at 4°C for 30 min. Uniform Manifold Approximation and Projection (UMAP) analysis was performed on manually gated CD8^+^ T cells, using 5,000 events per sample, 15 neighbors, and a minimum distance of 0.5. The following markers were included for UMAP generation: CD28, CCR7, CX3CR1, CXCR4, CXCR2, CXCR6, CCR2, and CCR5. Unsupervised clustering was then conducted with the FlowSOM algorithm (grid size: 10 × 10) using the same markers, resulting in five distinct clusters.

Cells were acquired on a NovoCyte Quanteon (Agilent). Gating was guided by FMO controls and analyzed with Novoexpress v1.5.0 (Agilent) or FlowJo v10 (Tree Star).

### 2.6 ELISA

Serum samples were thawed and analyzed immediately using the 2-CAT ELISA Fast Track kit for Norepinephrine and Epinephrine (LDN). Absorbance at 450nm wavelength was determined using Epoch plate reader and analyzed with Gen5 Take3 software (both Agilent). Concentrations were interpolated from standard curves according to the manufacturer’s protocol.

### 2.7 ELISPOT

Thawed PBMCs were rested overnight in X-Vivo 15/5% human serum. 3–5 × 10^5^ PBMCs were plated in IFN-γ Ab-coated ELISPOT plates and stimulated in triplicates with 5 µM of GLCTLVAML (EBV, Schafer-N) peptide for 20–24 h. After washing, biotinylated IFN-γ Ab, streptavidin-AP, and BCIP/NBT substrate (all Mabtech) were applied sequentially. Spots were analyzed on a CTL ImmunoSpot S6 Ultimate-V using ImmunoSpot v5.1 (both ImmunoSpot). Responses were normalized to 5 × 10^5^ PBMCs and reported as the difference between average numbers of spots in wells containing peptides and negative control wells.

### 2.8 DNA-barcoded virus peptide-MHC I multimer assay

Peripheral blood mononuclear cells (PBMCs) were screened for virus-reactive CD8^+^ T cells (VARTs) using a panel of 250 unique HLA-A and B restricted peptides from 20 different viruses, which were incorporated into barcode-labeled pMHC-multimer complexes (26). The peptides are listed in **Supplementary Table S2**. Specifically, pMHCs and unique DNA barcodes were attached to Phycoerythrin (PE) fluorochrome-labeled dextran molecules (Fina Biosolutions), generating unique DNA barcode-labeled pMHC I multimers for each pMHC combination. The cells were then stained with a combination of these multimers, along a phenotype antibody panel including CD3, CD8, CD45RA, CCR7, CD57, CD95 and PD-1 (**Supplementary Table S1**). PE-labeled CD8^+^ T cells were subsequently sorted using a FACSAria flow cytometer (BD Biosciences). DNA barcodes from the sorted cells were amplified via PCR, along with a reference DNA barcode baseline sample from the pMHC multimer panel used for staining. The amplified barcodes, including those from the sorted cells and the baseline, were sequenced by PrimBio. The sequencing data were then uploaded to Barracoda for analysis (26), including additional information on primers, DNA barcodes, pMHC barcode annotations, and sample identification. Analysis was performed with FlowJo v10.

### 2.9 Data analysis

This study included all samples (n = 23 participants; timepoints bsl, ex02, ex60), unless otherwise stated. As this was an exploratory study, no formal power calculations were performed. Analyses were advised by two independent bioinformaticians/data scientists. Data were analyzed and visualized using GraphPad Prism v10 or R v4.3.0 with RStudio 2024.04.2 + 764, Figures were created in Inkscape 1.3.2. Normality was assessed by histogram inspection or Shapiro-Wilk test. Significance between three groups was tested using one-way ANOVA, unless otherwise stated. Continuous variables are presented as mean ± SD unless otherwise noted. Correlations were performed using Spearman’s method unless otherwise specified. Multiple-testing correction was applied for appropriate ANOVA *post hoc* analyses and for correlation analyses summarized in heatmaps (Holm adjustment). No correction was applied across figures or panels, as these represent independent, pre-specified biological endpoints. Further details are provided in figure captions.

## 3 Results

### 3.1 INHALE study design, exercise-induced T cell response, and its correlation with catecholamine dynamics and individual factors

A total of 24 HLA-A2+ participants were included in the INHALE study, of which 23 participants conducted the full exercise intervention, samples and tests (see study design in **Figure 1A**). Participants had a mean age of 37.3 years (25–65), with 43.5% females, further characteristics are presented in **Table 1**. No adverse events occurred. VO2 peak (25) reflected a healthy cohort with variable fitness levels (**Supplementary Figures S1A, B**), and maximal power output and HR relative to the maximal values at CPET confirmed high exercise intensity during HIIT (**Supplementary Figures S1C-F**). Analysis of peripheral blood taken as part of the acute HIIT confirmed profound mobilization of T cells, which increased 2.0-fold (1.5-2.9, p<0.0001) (**Supplementary Figure S1G**), and CD8+ T cells, which increased 2.6-fold (1.8-4.1, p<0.0001) (**Figure 1B**). At ex60, counts of CD8+ T cells declined below baseline (**Figure 1B**). We also observed that higher CD8+ than CD4+ mobilization lowered the CD4/CD8 ratio at ex02, with a subsequent rise at ex60 (**Supplementary Figure S1H**). Interestingly, both catecholamines norepinephrine (NE) (**Supplementary Figure S1I**) and epinephrine (EPI) peaked at ex02 (**Supplementary Figure S1J**), however CD8+ mobilization selectively correlated with NE (R = 0.531, p= 0.0091) (**Figure 1C**), but not with EPI (R = 0.097, p=0.66) (**Figure 1D**). Notably, robust CD8+ T cell mobilization was observed consistently across participants regardless of VO2 peak, sitting time, BMI, lean body mass, body fat mass, or alcohol consumption (**Figure 1E**) and did not differ between male and female participants. Also, mobilization occurred independent of exercise parameters (**Supplementary Figure S2A**). This held also true for CD8+ T cell egress, which was independent of individual factors (**Supplementary Figure S2B**). Overall, we only observed a correlation between CD8+ T cell mobilization and NE.

### 3.2 Memory subsets and expression of differentiation and senescence markers on exercise-mobilized CD8+ T cells, and the influence of participant characteristics

**Figure 2:**
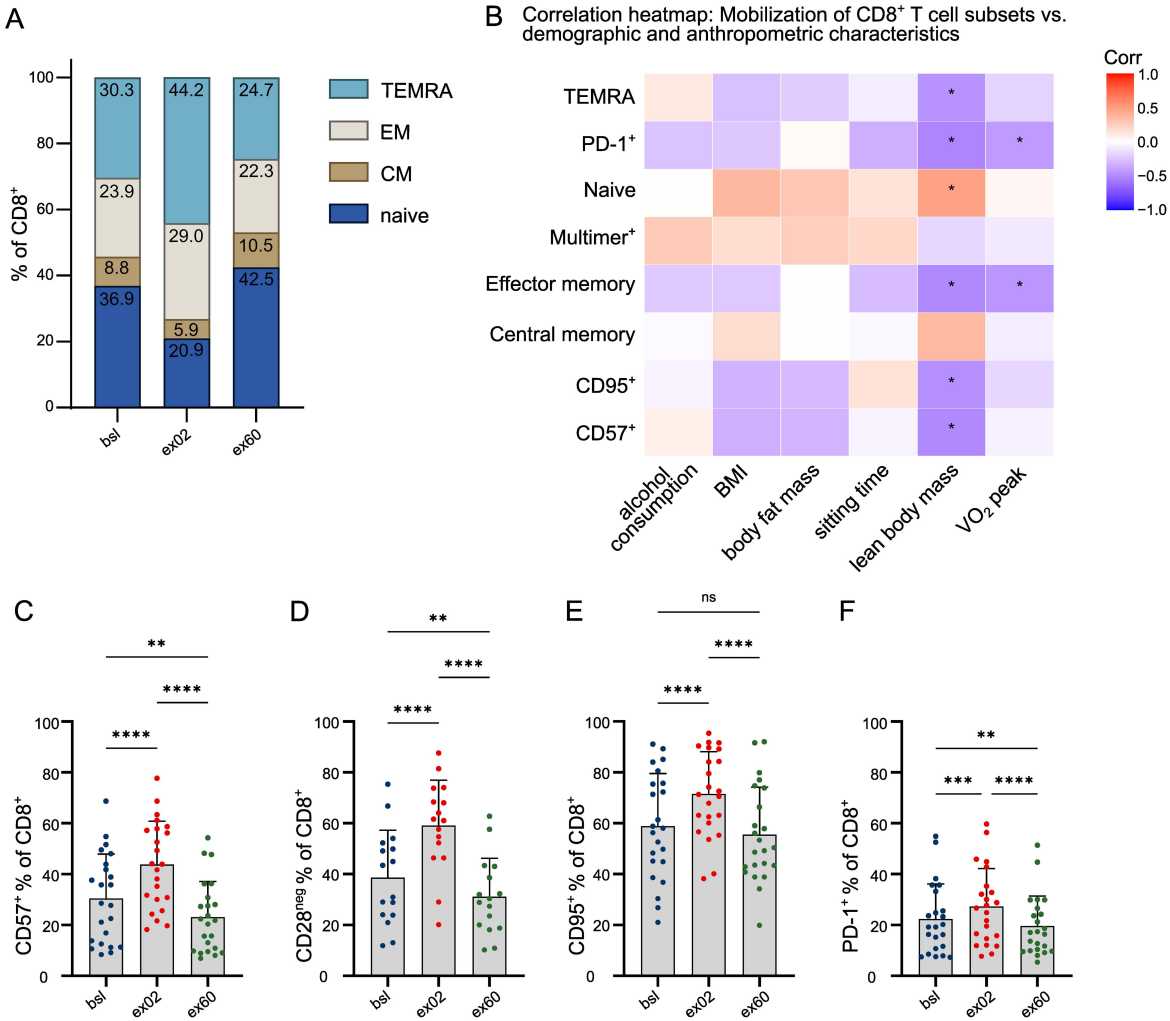
Acute HIIT changes the CD8^+^ T cell compartment’s memory subsets and exhaustion status. **(A)** Mean percentages of CD8^+^ memory subsets based on expression of CCR7 and CD45RA analyzed by flow cytometry. **(B)** Age-adjusted correlation heatmap of exercise-induced differences of phenotypic CD8^+^ subset frequencies (logit ex02/bsl) as assessed by flow cytometry vs. demographic, anthropometric and fitness participant characteristics. Unmarked correlations did not reach statistical significance. **(C-F)** Circulating CD8^+^ T cells were gated as **(C)** CD57^+^, **(D)** CD28^neg^, **(E)** CD95^+^ and **(F)** PD-1^+^. Results are presented as individual values per participant, and bars representing mean and SD. n=23; Significance levels indicated by asterisks on the graphs: ns p > 0.05, *p ≤ 0.05, **p ≤ 0.01, ***p ≤ 0.001, ****p ≤ 0.0001; CM central memory, EM effector memory, TEMRA terminally differentiated effector memory T cells.

We characterized the exercise-mobilized CD8+ T cells by their differentiation status (27, 28). The composition of memory subsets based on staining with CD45RA and CCR7 (28) shifted at ex02 in favor of terminally differentiated effector memory (TEMRA) (p<0.0001) and effector memory (EM) (p<0.0001) with a concomitant decrease in naïve CD8+ T cells (p<0.0001) and central memory (CM) (p<0.0001) (**Figure 2A**). CD8+ T cell mobilization strongly correlated with the mobilization of TEMRA and EM subsets, suggesting that they constitute the majority of exercise-responsive CD8+ T cells (**Supplementary Figures S2C, D**). At ex60, naïve CD8+ T cells had increased beyond bsl (p=0.0330) as did CM (p=0.0096) accompanied by decrease in TEMRA (p=0.0030) (**Figure 2A**). Investigation of the association between mobilization of these subsets and individual participant factors revealed that VO2 peak was inversely correlated with mobilization of EM and PD-1+ CD8+ T cells (**Figure 2B**). To this end, lean body mass positively correlated with mobilization of naïve CD8+ T cells, while a negative correlation was observed with mobilization of TEMRA, EM, CD57+, CD95+ and PD-1+ CD8+ T cells (**Figure 2B**). In line with the memory subset dynamics, one acute HIIT induced a higher percentage of CD57+ (p<0.0001) (**Figure 2C**), CD28neg (p<0.0001) (**Figure 2D**), CD95+ (p<0.0001) (**Figure 2E**) and PD-1+ (p=0.0001)(**Figure 2F**) circulating CD8+ T cells, representing higher percentages of T cells expressing markers of late differentiation, indicative of both senescent (CD57+, CD28neg) and exhausted (CD95+, PD-1+) CD8+ T cells phenotypes in the peripheral blood (summarized in **Supplementary Figure S2E**). This increase was transient, resulting in lower percentages of CD57+ (p=0.0050), CD28neg (p=0.0055) and PD-1+ (p=0.0040) CD8+ T cells at ex60 compared to bsl (**Figures 2C, D, F**). In addition to the percentage, the median fluorescence intensity (MFI) of PD-1+ CD8+ T cells was decreased (**Supplementary Figure S2F**). This suggests, that acute HIIT induces transient mobilization of late-differentiated CD8+ T cells followed by a recovery of naïve and less-differentiated cells above bsl.

### 3.3 Quantification of exercise-induced virus-specific CD8^+^ T cells (VARTs)

**Figure 3:**
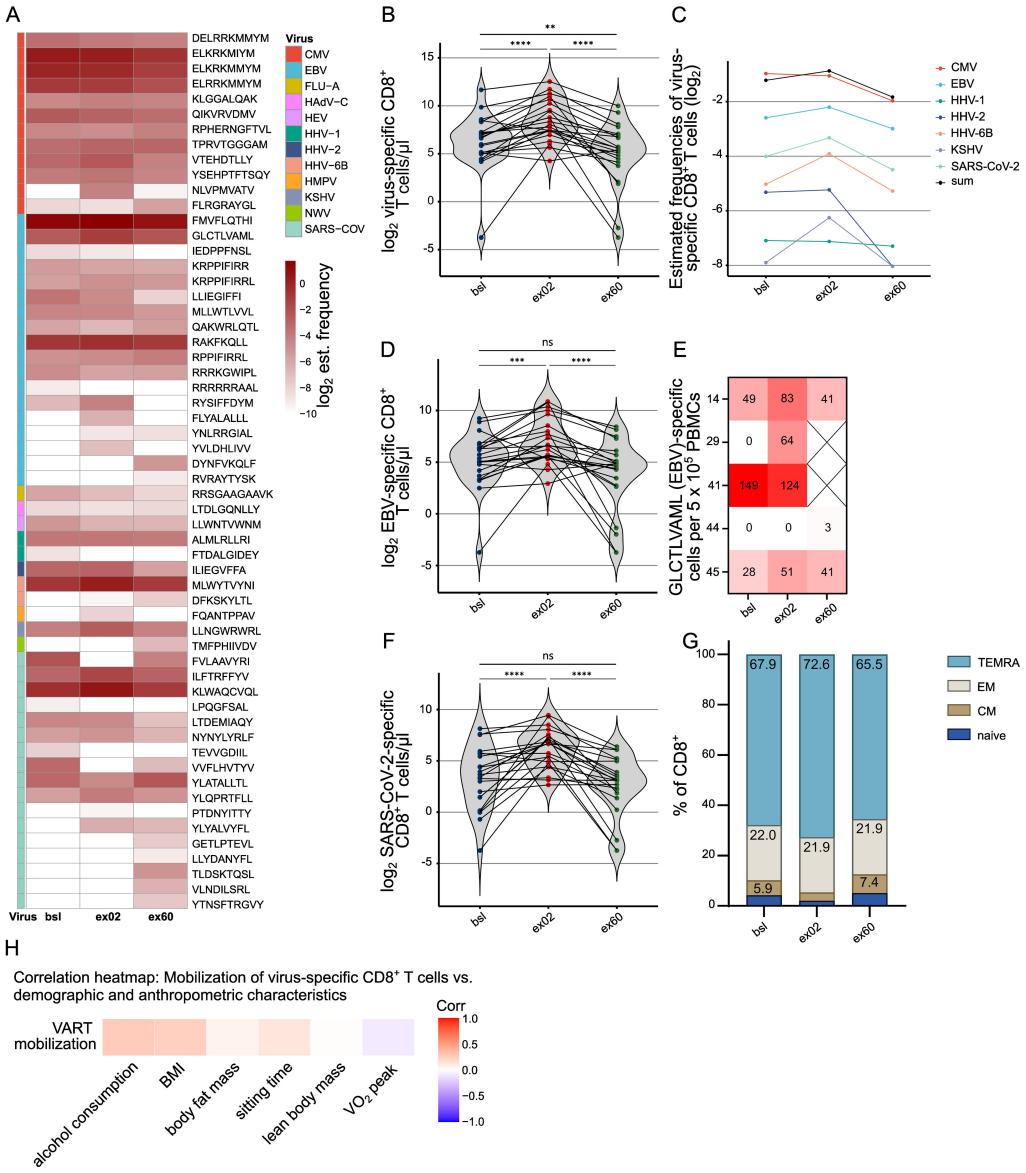
Acute HIIT selectively increases circulating virus-reactive CD8^+^ T cells, regardless of virus type. **(A)** Heatmap showing the estimated frequencies of peptide-specific CD8^+^ T cells per peptide. Analysis by DNA barcode-labeled peptide-MHC multimers, grouped after the respective virus. Darker colors indicate higher frequencies of peptide-specific CD8^+^ T cells. **(B)** Number of virus peptide-specific CD8^+^ T cells reactive against any of the 250 virus peptides. The estimated frequencies of MHC-matched, single peptide-specific CD8^+^ T cells were summed and multiplied by the absolute count of CD8^+^ T cells measured using the TBNK kit. **(C)** Estimated VART frequencies, summarized per virus. Plotted is the mean across all participants with a response at any timepoint on a log2 scale. **(D)** Numbers of EBV-specific CD8^+^ T cells against any EBV peptide, calculated according to **(B, E)** IFN-γ response to EBV peptide (GLCTLVAML). Plot shows the number of IFN-γ secreting cells per 5 x 105 PBMCs. ELISPOTS were run in technical triplicates. Spot counts are given as a difference between averages of the wells stimulated with the peptide and control wells. Samples were selected based on availability (n=5). **(F)** Numbers of specific CD8^+^ T cells against any SARS-CoV-2 peptide, calculated according to **(B, D, F)** Individual values overlaid with violin plots are plotted. P-values were calculated with Wilcoxon test and are Holm adjusted. **(G)** Proportions of CD8^+^ multimer^+^ memory subsets based on flow cytometric CCR7 and CD45RA staining. (n=23) **(H)** Age-adjusted correlation heatmap of exercise-induced VART mobilization (defined as log2 (VART ex02/bsl)) vs. demographic, anthropometric and fitness participant characteristics. No correlations reached statistical significance. HLA genotyping revealed 22 participants matching the HLA restrictions of the peptide pool, thus n=22 participants are analyzed for panels **(A-F, H)** Significance levels indicated by asterisks on the graphs: ns p > 0.05, *p ≤ 0.05, **p ≤ 0.01, ***p ≤ 0.001, ****p ≤ 0.0001. EBV Ebstein Barr virus, HLA human leukocyte antigen, MHC major histocompatibility complex.

To investigate the impact of acute HIIT on virus-reactive T cells (VART) a large-scale analysis was performed using DNA barcode-labeled virus peptide-MHC multimers (26) (peptide and derived virus list in **Supplementary Table S2**). Representative stainings of virus multimer-binding T cells are shown in **Supplementary Figure S3A**. Overall, VARTs were detected for 58 peptides derived from 12 viruses with consistent exercise-induced mobilization patterns across all viruses, both latent herpesviruses or lytic viruses (**Figure 3A**). First, the number of VARTs recognizing one or more virus peptides was analyzed: Exercise-mobilized VARTs significantly increased at ex02 (p<0.0001) and declined below baseline at ex60 (ex60 vs. ex02 p<0.0001, ex60 vs. bsl p=0.0090) (**Figure 3B**). To discriminate the predominant mobilization of VARTs from a purely quantitative mobilization effect, we analyzed the estimated frequencies, which confirmed preferential mobilization of VARTs following acute HIIT (**Figure 3C**). We then quantified VARTs for the two viruses being reactive in the most participants (**Supplementary Table S3**) and at the same time the highest number of recognized peptides (**Figure 3A**): EBV and SARS-CoV-2. 21 participants exhibited quantifiable EBV peptide-specific CD8+ T cells at bsl, 22 at ex02, and 20 at ex60 (**Supplementary Table S3**) in response to a total of 19 EBV-derived peptides (**Figure 3A**). Acute HIIT significantly mobilized EBV-reactive CD8+ T cells (p=0.0003), followed by a marked decline (ex60 vs. ex02 p<0.0001) (**Figure 3D**). The findings were supported by IFN-γ ELISPOT analysis to the dominant EBV peptide and showed mobilization of a highly functional CD8+ subset capable of IFN-γ secretion: 2 participants showed enhanced, 1 showed an IFN-γ response only detectable after exercise (ex02 or ex60), 1 participant showed a strong response at both bsl and ex02 (ex60 sample not available), and 1 participant showed no response (**Figure 3E**). SARS-CoV-2 peptides elicited responses in 21 participants at bsl, 22 at ex02, and 20 at ex60 (**Supplementary Table S3**), frequencies (**Figure 3C**) and cell counts were minimally lower compared to EBV (**Figure 3F**). Memory subset analysis of multimer^+^ CD8^+^ T cells at bsl revealed a predominance of TEMRA and EM with minor CM and naïve fractions (**Figure 3G**). Overall, acute HIIT induced only minor shifts reflecting a homogeneous multimer+ CD8+ T cell population: At ex02, a shift toward TEMRA (p=0.0003) with reduced naïve (p=0.0002) and CM (p=0.0110) mirrored the pattern of total CD8^+^ T cells but was less pronounced. At ex60, CM had increased compared to bsl (p=0.0345) while other subsets remained largely unchanged (**Figure 3G**). Furthermore, the fractions of CD95+ and PD-1+ were unchanged by acute HIIT, while the CD57+ population was reduced at ex60 vs. ex02 (p<0.0001) (**Supplementary Figures S3B-E**). The mobilization of VARTs occurred independent of individual demographic, anthropometric and cardiorespiratory fitness parameters (**Figure 3H**).

### 3.4 Expression of chemokine receptors and their cognate ligands

**Figure 4:**
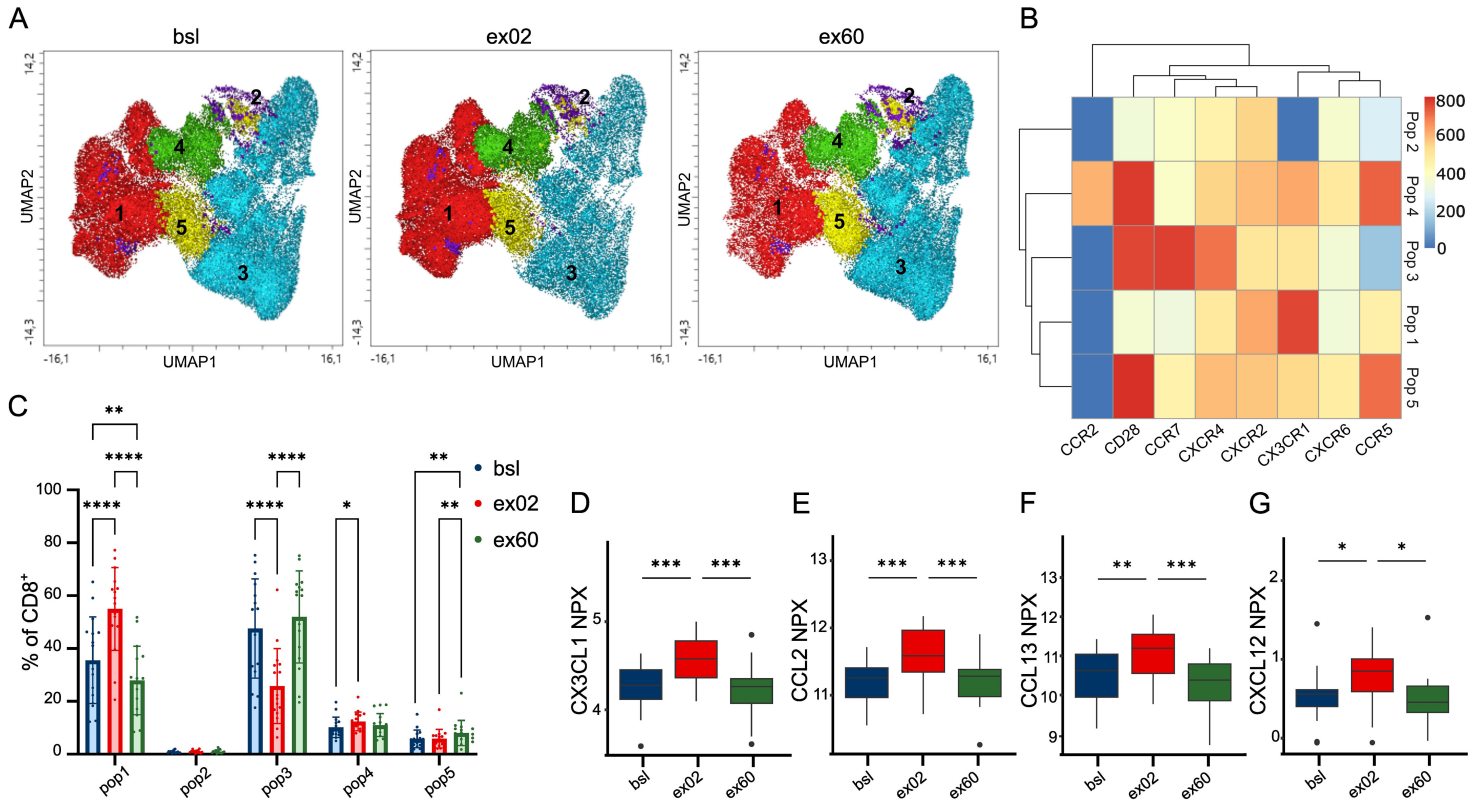
Exercise-induced modulation of chemokine receptors and their ligands suggests enhanced capacity for CD8^+^ T cell migration, activation and memory formation. **(A)** UMAP visualizing the clusters identified via the FlowSOM unsupervised clustering algorithm on live CD8^+^ T cells at bsl, ex02 and ex60. Clustering was based on the expression of chemokine receptors CCR5, CXCR2, CXCR6, CX3CR1, CCR2, CXCR4, as well as CD28 and CCR7. **(B)** Heatmap showing the surface expression profiles of the 5 identified clusters. **(C)** Relative population frequency at bsl, ex02 and ex60. Shown are individual values, and bar graphs showing mean and SD. **(A-C)** Results from n=16 participants are presented. **(D-G)** Serum levels of those 4 cognate ligands of the analyzed chemokine receptors that were significantly regulated at ex02 compared to bsl. Box-and-whisker-plots representing median and the 25th to 75th percentiles of normalized protein expression (NPX) values from n=23 participants. **(D)** CX3CL1, **(E)** CCL2, **(F)** CCL13 and **(G)** CXCL12. Significance levels indicated by asterisks on the graphs: *p ≤ 0.05, **p ≤ 0.01, ***p ≤ 0.001, ****p ≤ 0.0001.

Finally, after mobilization of CD8+ T cells, their cytotoxic functionality also depends on egression to relevant tissues. To this end, we investigated if acute HIIT causes a dynamic shift in chemokine receptor patterns of the circulating CD8+ T populations (**Figure 4A**). We identified CD8+ CD28neg CCR7neg T cells, corresponding to late-differentiated antigen-specific CD8+ T cells (29), which are characterized by uniquely high expression of CX3CR1 and medium expression of CXCR2 (pop1) to be preferentially mobilized by acute HIIT before decreasing below bsl (**Figures 4B, C**). Population 4 (pop4), a CD8+ CD28+ CCR7neg T cell population characterized by high CCR5 expression with medium CX3CR1, CXCR2 and unique CCR2 expression, is preferentially mobilized (**Figures 4B, C**) and corresponds to a population in the process of differentiation with enhanced tissue homing capacities (29). In contrast, the percentage of population 3 (pop3) as characterized by CD28+ CCR7+ CXCR4+ and otherwise low chemokine receptor expression below median (CCR2, CXCR2, CX3CR1, CXCR6 and CCR5) corresponds to a naive or early-differentiated population, decreased at ex02 and was above bsl at ex60 (**Figures 4B, C**). At ex60, a CD8+ CD28+ CCR7low population (pop5) had increased above bsl frequencies. Single UMAP plots illustrating the expression of distinct chemokine receptors are shown in **Supplementary Figures S4A-H**. Changes of the frequencies of individual circulating chemokine receptor-positive CD8+ T cells are shown in **Supplementary Figures S4I-N**. Elevated MFI of CXCR2 and CXCR6 suggests an increased chemotactic sensitivity (**Supplementary Figures S4O-T**). Age is the only variable influencing any of the chemokine receptor expressing subsets (data not shown). Chemokines specific to the analyzed receptors were strongly regulated by acute HIIT: Proteomic analyses revealed regulation of 9 of 11 analyzed ligands by acute HIIT, with 4 being significantly increased at ex02: CX3CL1 (**Figure 4D**), CCL2 (**Figure 4E**), CCL13 (**Figure 4F**) and CXCL12 (**Figure 4G**). These data show that exercise modifies chemokine receptor expression on CD8+ T cells and their ligands, suggesting a potential impact on the migratory capacity of immune cells.

## 4 Discussion

Frequent physical activity significantly modulates the immune system and its ability to respond to harmful or foreign antigens, but the underlying mechanisms remain not well-defined, and comprehensive studies are lacking. Earlier studies are limited to isolated aspects of exercise immunology and are conducted in a variety of exercise settings regarding FITT criteria, or non-human species (13, 30). Expanding on current work, we systematically investigated individualized CD8+ T cell responses to acute HIIT, encompassing adrenergic signaling, memory subset dynamics, virus specificity to a broad panel of viral antigens and chemokine receptor-ligand profiles a well-characterized cohort of 23 healthy individuals under strictly controlled conditions. We minimized circadian effects by scheduling exercise within a 5-hour daylight window (31).

Firstly, we showed that all participants robustly mobilized CD8+ T cells. The magnitude of CD8+ T cell mobilization (2.6-fold) was higher than previously reported by Anane et al. (~2.0-fold) (32) and Campbell et al. (~2.4-fold; based on pre and post means) (12), likely reflecting differences in exercise regimens and methodological rigor: In our study, blood was collected within 2 minutes post-HIIT and processed using clinically validated protocols, ensuring reliable immune cell quantification. These sampling timepoints have since proven to catch meaningful exercise responses in the CD8+ T cell compartment and reflect both acute and the redistribution effects (33). We identified mobilization of CD8+ T cells to be independent of individual characteristics, suggesting benefits for a broad range of individuals. While exercise-induced leukocytosis is mainly mediated by catecholamines via adrenergic receptor signaling (10), our observation of a dominant association of NE with CD8+ T cell mobilization is novel. EPI and NE are secreted concomitantly, however, differ in receptor affinity and production sites. This would partly explain why CD8+ T cell tissue infiltration generally exceeds that of NK cells. In previous studies, EPI seemed to govern exercise effects, but explained only part of the effects (13, 34), supporting a necessary but not sufficient role for CD8+ T cell mobilization.

Secondly, we observed a striking reorganization of circulating CD8+ T cells indicative of preferential mobilization of antigen-experienced cells. This aligns with reports of predominant mobilization of late-differentiated CD8+ T cells (9, 35–37), while such a broad phenotyping has not been applied to explore T cell egress. Given that EM and TEMRA cells predominantly drive CD8+ T cell mobilization in our study, NE may preferentially mobilize these subsets, while EPI could act more on naïve or CM cells. This would align with recent work showing that phosphodiesterase-4 (PDE4) inhibition enhances exercise-induced mobilization of less responsive subsets via β-adrenergic signaling (38). Our findings differ from previous reports linking circulating CD95+ CD8+ T cells with body fat (39), raising questions about whether muscle might mediate beneficial immune effects while fat could promote detrimental immune effects in the context of exercise. We furthermore demonstrate that acute exercise markedly increases both the relative frequency and absolute number of virus-specific CD8+ T cells reactive against peptides derived from latent herpes- and lytic viruses (26). While individual vaccination histories were not available, participants were healthy adults and thus likely adhered to the Danish vaccination program (a primary two-dose SARS-CoV-2 vaccination early 2021, influenza vaccination optional). However, potential effects of recent vaccination on magnitude and phenotype of vaccine-reactive T cell mobilization cannot be excluded. While mobilization has previously been shown for CMV-, EBV- (20), SARS-CoV-2- (9, 40) and adenovirus-specific (41) T cells using peptide mixes and ELISPOT only or TCRseq-based predicted specificities, our study took advantage of DNA-barcoded peptide-MHC multimers (36, 42). Exercise has been shown to recruit Cytomegalovirus (CMV)- (43), Ebstein Barr Virus (EBV)- (20), SARS-CoV-2- (9, 40) and Adenovirus (HAd)-specific (41) T cells into peripheral blood. The magnitude of SARS-CoV-2-specific CD8+ T cell mobilization in our study is in line with previously described values (9). Importantly, our multimer-based analysis spanning the timeline from bsl to one hour post-HIIT not only confirms these findings, but extends them by showing a substantial egress one hour post-HIIT and by demonstrating this redistribution effect for 12 different viruses. Spielmann et al. reported increased antigen response, and superior expansion and enhanced functionality compared to T cells obtained in a resting state (20). However, knowledge of temporal dynamics regarding diversity and relative frequency of antigen-specific T cells following exercise, as well as the influence of virus type on virus-reactive T cell (VART) responses, remains incomplete.

This raises the question if CD8+ T cell mobilization or redistribution are crucial for enhancing tissue immunity. Walsh et al. suggest that these egressing cells migrate to effector tissues and subsequently re-enter the blood via the lymphatic system (44). Wu et al. further evidenced that circulating T cell clones can also be found in the tissue, thereby exerting local immunosurveillance (45) and supporting the importance of lymphocyte egress.

The third aim of this study revealed a shift in CD8+ T cell migratory and functional potential based on the chemokine receptor expression pattern: Importantly, HIIT mobilizes not only terminally differentiated but also transitional CD8+ T cell populations with enhanced migratory capacity. The highest relative mobilization corresponded to a population of circulating late-differentiated CD8+ T cells characterized by high CX3CR1 and medium CXCR2 expression. Chemokine receptor CX3CR1 is associated with potent cytotoxicity and its overexpression on CAR T cells has been demonstrated to promote infiltration into the TME (46, 47). Distinct exercise-mobilized CD8+ T cell subsets were characterized by enhanced tissue migration properties, specifically CCR2, CCR5 and CXCR2 (48, 49). The concomitantly increased chemokine levels might support CD8+ T cell trafficking to effector tissues and enhance their cytotoxic capacity, thereby potentially optimizing antiviral immunity.

Limitations of our study include unavailable CMV serostatus in our cohort. CMV+ individuals have been shown to have a higher percentage of highly differentiated and senescent T cell subsets, and thus CD8+ T cell response to exercise might be different (43, 50). The second limitation is the HLA-A2 restriction of our study cohort to optimize detection of virus-reactive CD8+ T cells by the applied pMHC multimer panel. Even though this is the most abundant HLA type worldwide, it might limit generalizability, since certain viral epitopes are presented by other HLA alleles than A2 and virus-specific CD8+ T cell responses to exercise could differ in individuals with other HLA types.

Taken together, our findings support exercise as a low-cost, modifiable strategy to enhance T cell immunity, even with brief, intense activity in a broad range of individuals, irrespective of demographics, anthropometrics or fitness. They also raise critical questions for patient care: Can regular high-intensity exercise reshape cellular immunity? Does HIIT improve immune cell infiltration in the tumor microenvironment?

## Data Availability

The raw data supporting the conclusions of this article will be made available by the authors, without undue reservation.
