# Supplementary material for: Moving for optimal immunity: the effect of acute high-intensity interval training on phenotype, virus specificity and chemokine receptor expression in human CD8+ T cells
Source: Front Immunol. 2026 Jan 19;16:1739657. doi: 10.3389/fimmu.2025.1739657 (PMC12888222; doi:10.3389/fimmu.2025.1739657)
Supplement: Supplementary file 1 [file DataSheet1.pdf]

Supplementary Material

Moving for optimal immunity: The effect of acute high-intensity interval training on phenotype, virus specificity and chemokine receptor expression in human CD8+ T cells

Katharina Leuchte[1,2*], Thy Viet Luu[1], Sara Fresnillo Saló[1], Kasper Madsen[1], Lise Heide-Ottosen[3], Signe Koggersbøl Skadborg[4], Janine Sophie Kemming[4], Morten Orebo Holmström[1], Hongjin Chen[5], Lars Rønn Olsen[5,6], Anders Vinther[7,8], Mads Hald Andersen[1], Sine Reker Hadrup[4], Per thor Straten[1#], Gitte Holmen Olofsson[1#]

[1] National Center for Cancer Immune Therapy (CCIT-DK), Department of Oncology, Copenhagen University Hospital Herlev, 2730 Herlev, Denmark.

[2] Department I of Internal Medicine, Medical Faculty and University Hospital of Cologne, University of Cologne, 50937 Cologne, Germany.

[3] Department of Oncology, Copenhagen University Hospital Herlev, 2730 Herlev, Denmark.

[4] Department of Health Technology, Section of Experimental and Translational Immunology, Technical University of Denmark, 2800 Kongens Lyngby, Denmark.

[5] Department of Health Technology, Bioinformatics, Single Cell Omics, Technical University of Denmark, 2800 Kongens Lyngby, Denmark.

[6] Department of Immunology and Microbiology, LEO Foundation Skin Immunology Research Center, University of Copenhagen, 2200 Copenhagen, Denmark.

[7] Department of Physiotherapy and Occupational Therapy, Copenhagen University Hospitals Herlev and Gentofte, 2730 Herlev and 2900 Hellerup, Denmark.

[8] Department of Clinical Medicine, University of Copenhagen, 2200 Copenhagen, Denmark.

[#] These two authors contributed equally to this work.

***Correspondence:**
Katharina Leuchte, e-mail: katharina.leuchte@regionh.dk

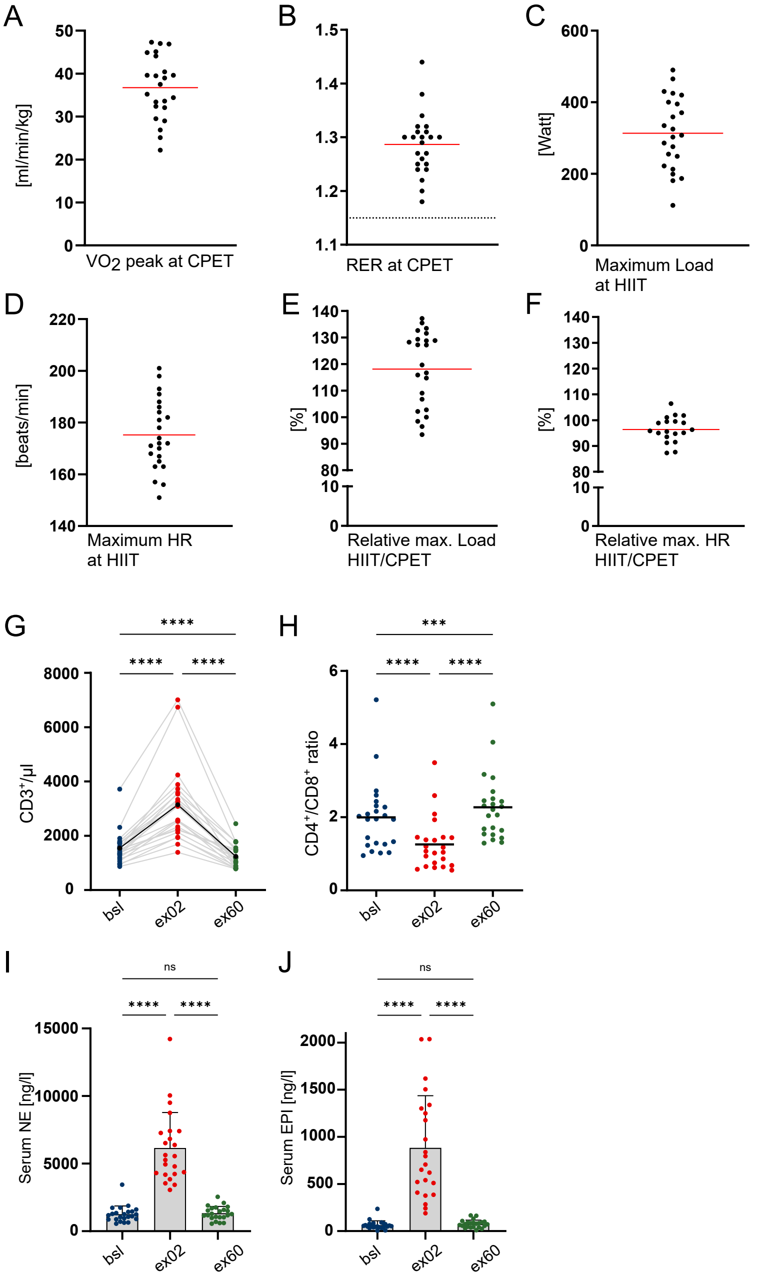

Figure S1. High-intensity exercise induced T cell mobilization and a shift of CD4/8 ratio, and peak of serum catecholamines. A) VO_2 peak with B) corresponding RER as determined in the CPET (visit 2). C) Maximum power output and D) maximum heart rate in the HIIT exercise session (visit 3). E) Relative maximum power output and F) relative maximum heart rate in the exercise session divided by these values at CPET. A-F) Individual values (n=23) per participant are shown. Red line indicates mean. G, H) Acute HIIT-induced cell count dynamics using flow cytometric TBNK kit whole blood staining of G) $CD3^+$ T cells, and H) CD4/CD8 ratio. I) Norepinephrine (NE) and J) Epinephrine (EPI) serum levels before (bsl), immediately after (ex02) and one hour after exercise (ex60) determined by ELISA. Shown are individual values, and bars representing mean and SD. n=23; Significance levels indicated by asterisks on the graphs: ns $p > 0.05$, * $p \leq 0.05$, ** $p \leq 0.01$, *** $p \leq 0.001$, **** $p \leq 0.0001$. CPET cardiopulmonary exercise test, VO_2peak peak oxygen consumption, RER relative exchange ratio, HIIT High-intensity interval training, NE norepinephrine, EPI epinephrine

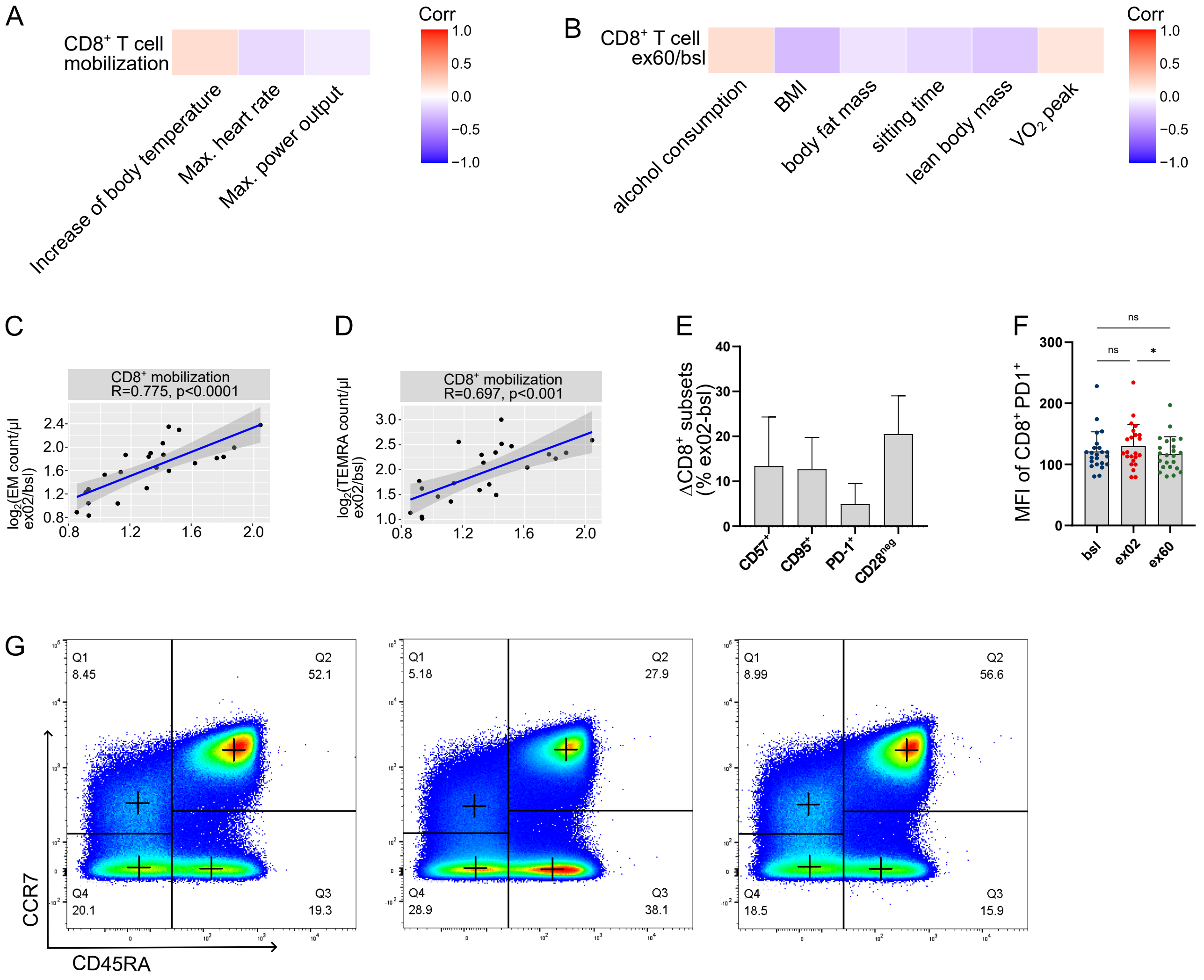

Figure S2. A) Age-adjusted correlation heatmap of CD8+ T cell reduction [log2(ex60/bsl)] vs. demographic and anthropometric participants' characteristics as assessed at inclusion or CPET. B) Age-adjusted correlation heatmap of immune cell mobilization (CD8+ T cells, NK cells) vs. vital parameters HR, maximum load and temperature increase. C, D) Scatterplots showing the strongest correlations between exercise-induced CD3+ CD8+ mobilization and phenotypic CD8+ subsets [log2(ex02/bsl)]. C) CD3+ CD8+ mobilization vs. TEMRA (CCR7- CD45RA+) mobilization. D) CD3+ CD8+ mobilization vs. Effector Memory (EM) (CCR7- CD45RA-) mobilization. [n=23] E) Exercise-induced increase of the respective CD8+ T cell proportions, calculated as difference of frequencies (ex02 – bsl), shown are mean and SD. F) MFI of PD-1+ CD8+ T cells. G) Representative density plots illustrating flow cytometric staining for CD45RA and CCR7 at bsl, ex02 and ex60. MFI = median fluorescence intensity. Significance levels indicated by asterisks on the graphs: ns p > 0.05, * p ≤ 0.05, ** p ≤ 0.01, *** p ≤ 0.001, **** p ≤ 0.0001.

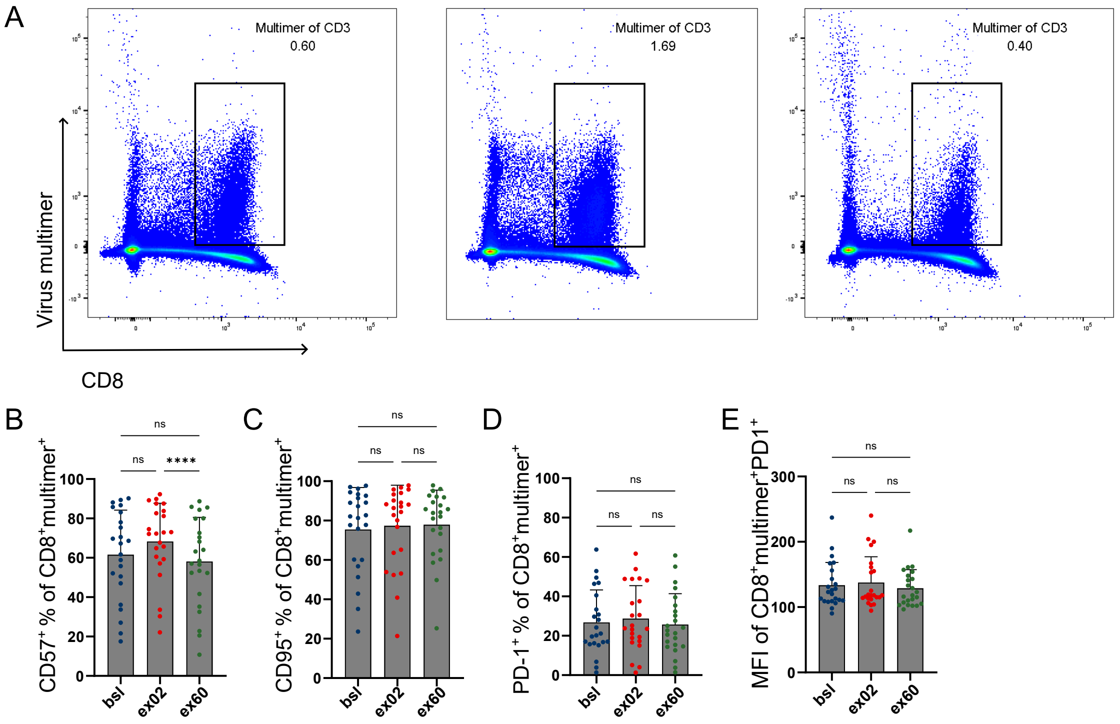

Figure S3. Phenotype of multimer+ CD8+ T cells. A) Representative density plots illustrating flow cytometric staining of multimer+CD8+ T cells at bsl, ex02 and ex60. B-E) Frequency of CD57+, CD95+ and PD-1+ fractions of CD8+ multimer+ T cells, and MFI of PD-1+ CD8+ multimer+ T cells. Significance levels indicated by asterisks on the graphs: ns $p > 0.05$, * $p \leq 0.05$, ** $p \leq 0.01$, *** $p \leq 0.001$, **** $p \leq 0.0001$.

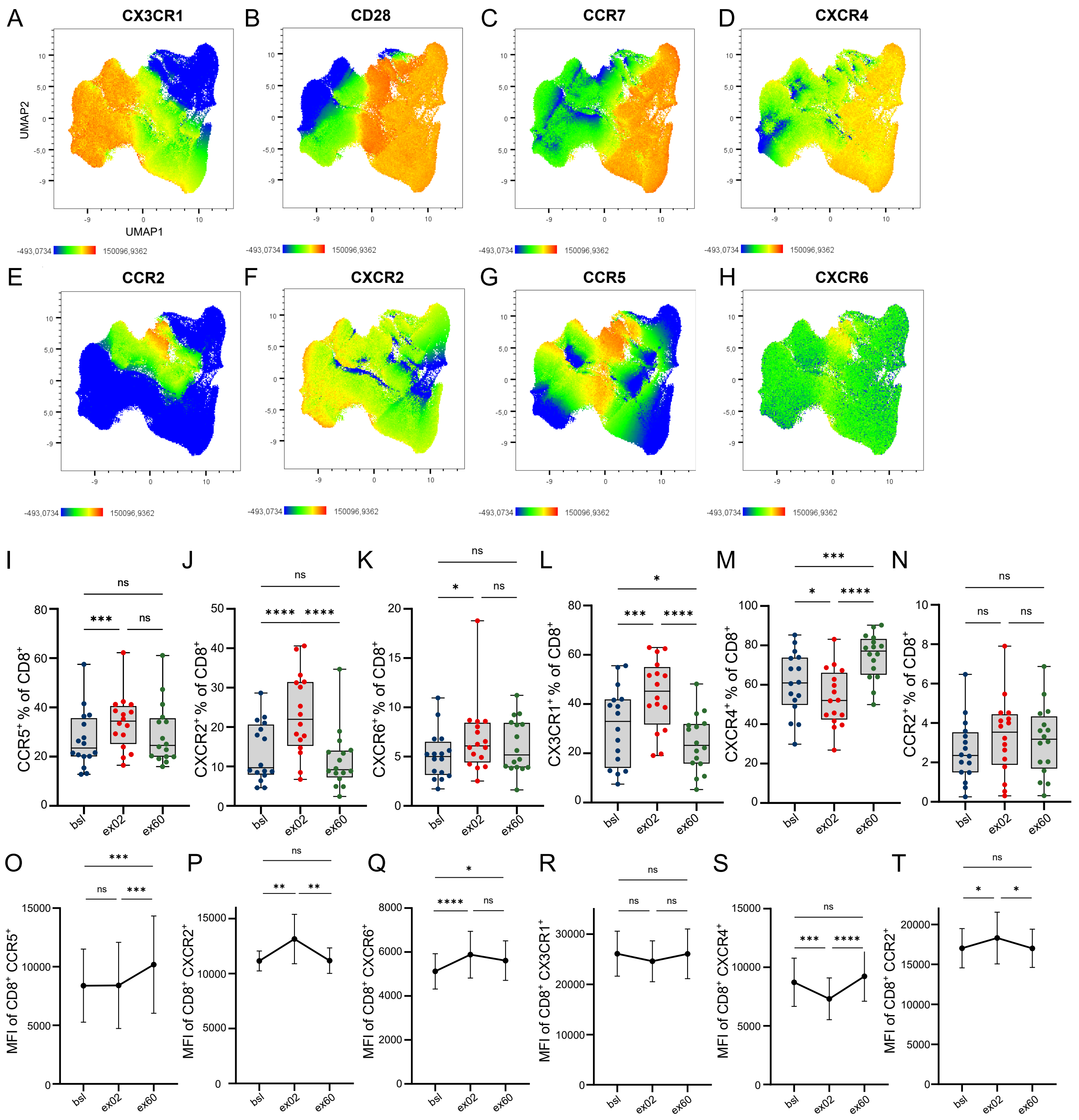

Figure S4. A-H) UMAP plots illustrating expression of distinct chemokine receptors. The UMAP plots were generated using pooled data of 240,000 cells from bsl, ex02 and ex60. A) CX3CR1, B) CD28, C) CCR7, D) CXCR4, E) CCR2, F) CXCR2, G) CCR5 and H) CXCR6. I-N) Frequency of chemokine receptor-expressing proportion of circulating $CD8^+$ T cells in percent. Plots show individual values per participant, and box-and-whisker plots representing median and the box extends from the 25th to 75th percentiles with whiskers from minimum to maximum values. I) CCR5+, J) CXCR2+, K) CXCR6+, L) CX3CR1+, M) CXCR4+ and N) CCR2+ CD8+ T cells. O-T) Median fluorescence intensity (MFI) of the positive population per timepoint. Shown are mean and SD. n=16 participants. Significance levels indicated by asterisks on the graphs: ns $p > 0.05$, * $p \leq 0.05$, ** $p \leq 0.01$, *** $p \leq 0.001$, **** $p \leq 0.0001$

ANTIBODY	MANUFACTURER	IDENTIFIER	CLONE
HLA-A2-PE	BD Biosciences, Franklin Lakes, NJ, USA	Cat.-No. 558570	Clone BB7.2
CD3ε-BV510	BD Biosciences	Cat.-No. 563109	Clone UCHT1
Vδ2-BV650	BD Biosciences	Cat.-No. 743752	Clone B6
Near IR (NIR)-APC-Cy7	Thermo Fisher, Waltham, MA, USA	Cat.-No. L34994	
Multimer-PE	Laboratory of Sine Reker Hadrup	N/A	
CCR7-APC	Biolegend, San Diego, CA, USA	Cat.-No. 353214	Clone G043H7
CD8-BV480	BD Biosciences	Cat.-No. 566121	Clone RPA-T8
CD3-FITC	BD Biosciences	Cat.-No. 345764	Clone SK7
CD127-BV605	BD Biosciences	Cat.-No. 562662	Clone HIL-7R-M21
CD38-BUV737	BD Biosciences	Cat.-No. 612824	Clone HIT2
CD279 (PD-1)-PE-CF594	BD Biosciences	Cat.-No. 565024	Clone EH12.1
CD57-PECy7	Biolegend	Cat.-No. 393310	Clone QA17A04
CD95-BUV395	BD Biosciences	Cat.-No. 740306	Clone DX2
CD45RA-BV421	BD Biosciences	Cat.-No. 562885	Clone HI100
CD28-PE-CF594	BD Biosciences	Cat.-No. 562323	Clone CD28.2
CCR2-BV421	BD Biosciences	Cat.-No. 564067	Clone 48607
CCR5-BV605	BD Biosciences	Cat.-No. 563379	Clone 2D7/CCR5
CXCR2 (CD182)-RY586	BD Biosciences	Cat.-No. 753253	Clone 6C6
CXCR4-APC	BD Biosciences	Cat.-No. 560936	Clone 12G5
CXCR6-BB700	BD Biosciences	Cat.-No. 745882	Clone 13B 1E5
CX3CR1-BB515	BD Biosciences	Cat.-No. 565902	Clone 2A9-1
CD3ε-BV510	BD Biosciences	Cat.-No. 563109	Clone UCHT1
CD8-BV711	BD Biosciences	Cat.-No. 563676	Clone RPA-T8
BD Multitest™ 6-Color TBNK with Trucount Tubes	BD Biosciences	Cat.-No. 337166	
Interferon γ (IFNγ)-capture antibody (Ab)	Mabtech, Nacka Strand, Sweden	Cat.-No. 3420-3-1000	
Biotin-conjugated secondary antibody IFNγ	Mabtech	Cat.-No. 3420-6-1000	

Table S1. Antibody List

PEPTIDE	VIRUS	HLA	SEQUENCE	PROTEIN
1	CMV	A0101	TSDACMMTMY	VIE1
2	CMV	A0101	VTEHDTLLY	VPAP
3	CMV	A0101	YSEHPTFTSQY	PP65
4	EBV	A0101	ESEERPPTPY	LMP2
5	EBV	A0101	HSDYQPLGT	LMP2
6	FLU-A	A0101	CTELKLSDY	NP
7	HAdV-C	A0101	LTDLGQNLLY	CAPSH
8	HEV	A0101	ATEASNYAQY	ORF2_176
9	HEV	A0101	SSAGGQLFY	ORF2_389
10	HHV-1	A0101	ATDSLNNEY	TEG1
11	HHV-1	A0101	FTDALGIDEY	VP16
12	HHV-1	A0101	SALPTNADLY	VP16
13	HHV-1	A0101	YMESVFQMY	RIR1
14	HHV-2	A0101	ASDSLNNEY	TEG1
15	HHV1	A0101	LIDGIFLRY	gpL
16	SARS-COV	A0101	CTDDNALAYY	ORF1
17	SARS-COV	A0101	DTDFVNEFY	ORF1
18	SARS-COV	A0101	FTSDYYQLY	ORF3
19	SARS-COV	A0101	LTDEMIAQY	S
20	SARS-COV	A0101	PTDNYITTY	ORF1
21	SARS-COV	A0101	YTNSFTRGVY	S
22	B19	A0201	GLCPHCINV	NS1
23	B19	A0201	LLHTDFEQV	NS1
24	BKPyV	A0201	LLMWEAVTV	VP1
25	BKPyV	A0201	VIFDFLHCI	LT
26	CMV	A0201	GVLDAVWRV	US08
27	CMV	A0201	NLVPMVATV	PP65
28	CMV	A0201	YILEETSVML	VIE1
29	EBV	A0201	CLGGLLTMV	LMP2
30	EBV	A0201	FLYALALLL	LMP2
31	EBV	A0201	FMVFLQTHI	EBNA1
32	EBV	A0201	GLCTLVAML	ICP27
33	EBV	A0201	LLIEGIFFI	RIR2
34	EBV	A0201	MLLWTLVVL	LMP2
35	EBV	A0201	TLDYKPLSV	EAD
36	EBV	A0201	YLLEMLWRL	LMP1
37	EBV	A0201	YLQQNWWTL	LMP1
38	EBV	A0201	YVLDHLIVV	RTA
39	FLU-A	A0201	GILGFVFTL	M1
40	HAdV-C	A0201	LLDQLIEEV	E1A
41	HEV	A0201	FLSRVQTEI	ORF1_37
42	HEV	A0201	GLLDFALEL	ORF2_298
43	HEV	A0201	LLWNTVWNM	ORF1_1527
44	HEV	A0201	RLMTYLRGI	ORF1_339
45	HEV	A0201	SLAKFIPTI	ORF1_1334
46	HEV	A0201	VLYAAPLNPL	ORF2_154
47	HEV	A0201	YVSDTVTFV	ORF2_493
48	HHV-1	A0201	ALMLRLLRI	OBP
49	HHV-2	A0201	ILIEGVFFA	RIR2

50	HHV-6B	A0201	MLWYTVYNI	IE1
51	HMPV	A0201	FQANTPPAV	MATRX
52	JCPyV	A0201	ILMWEAVTL	Q91UM0
53	KSHV	A0201	LLNGWRWRL	K12
54	NWV	A0201	TMFPHIIVDV	CAPSD
55	RTV	A0201	TLLANVTAV	VP6
56	SARS-COV	A0201	ALSKGVHFV	ORF3
57	SARS-COV	A0201	ALWEIQQVV	ORF1
58	SARS-COV	A0201	FGDDTVIEV	ORF1
59	SARS-COV	A0201	FIAGLIAIV	S
60	SARS-COV	A0201	FLLPSLATV	ORF1
61	SARS-COV	A0201	FLPGVYSV	ORF1
62	SARS-COV	A0201	FLPRVFSAV	ORF1
63	SARS-COV	A0201	FVLAAVYRI	M
64	SARS-COV	A0201	GLTVLPPLL	S
65	SARS-COV	A0201	GMSRIGMEV	N
66	SARS-COV	A0201	HLMSFPQSA	S
67	SARS-COV	A0201	ILFTRFFYV	ORF1
68	SARS-COV	A0201	KIADYNYKL	S
69	SARS-COV	A0201	KLLEQWNLV	M
70	SARS-COV	A0201	KLPDDFTGCV	S
71	SARS-COV	A0201	KLWAQCVQL	ORF1
72	SARS-COV	A0201	LLLDRLNQL	N
73	SARS-COV	A0201	LLYDANYFL	ORF3
74	SARS-COV	A0201	RLQSLQTYV	S
75	SARS-COV	A0201	SMWSFNPET	M
76	SARS-COV	A0201	TLDSKTQSL	S
77	SARS-COV	A0201	VLNDILSRL	S
78	SARS-COV	A0201	VVFLHVTYV	S
79	SARS-COV	A0201	YLATALLTL	ORF1
80	SARS-COV	A0201	YLQPRTFLL	S
81	SARS-COV	A0201	YLYALVYFL	ORF3
82	VACV	A0201	CLTEYILWV	VC16
83	VACV	A0201	KVDDTFYYV	C7
84	VACV	A0201	YLYTEYFLFL	A25
85	CMV	A0301	KLGGALQAK	VIE1
86	CMV	A0301	RIKEHMLKK	IE1
87	CMV	A0301	TTVYPPSSTAK	pp150
88	EBV	A0301	RLRAEAQVK	EBNA3
89	EBV	A0301	RVRAYTYSK	RTA
90	HEV	A0301	ILRAWIRTTK	ORF1_235
91	HEV	A0301	MTYLRGISYK	ORF1_341
92	HEV	A0301	RLYSWLFEK	ORF1_409
93	SARS-COV	A0301	KCYGVSPTK	S
94	SARS-COV	A0301	KLFDRYFKY	ORF1
95	SARS-COV	A0301	KTFPPTEPKK	N
96	SARS-COV	A0301	KTIQPRVEK	ORF1
97	SARS-COV	A0301	QVVNVVTTK	ORF1
98	SARS-COV	A0301	RASANLAATK	S
99	SARS-COV	A0301	RMYIFFASFY	ORF1

100	SARS-COV	A0301	VTNNTFTLK	ORF1
101	SARS-COV	A0301	VVYRGTTTYK	ORF1
102	CMV	A1101	ATVQGQNLK	pp65
103	CMV	A1101	SVLGPISGHVLK	pp65
104	EBV	A1101	ATIGTAMYK	RTA
105	EBV	A1101	AVFDRKSDAK	EBNA4
106	EBV	A1101	IVTDFSVIK	EBNA4
107	EBV	A1101	SSCSSCPLSK	LMP2
108	FLU-A	A1101	KTGGPIYRR	NP
109	HEV	A1101	MTYLRGISYK	ORF1_341
110	HEV	A1101	RLYSWLFEK	ORF1_409
111	HEV	A1101	TTTAATRFMK	ORF2_342
112	SARS-COV	A1101	ASMPTTIAK	ORF1
113	SARS-COV	A1101	ATEGALNTPK	N
114	SARS-COV	A1101	KTFPPTEPK	N
115	SARS-COV	A1101	SAFAMMFVK	ORF1
116	SARS-COV	A1101	STFNVPMEK	ORF1
117	B19	A2402	FYTPLADQF	NS1
118	CMV	A2402	MYMCYRNIEF	VIE1
119	CMV	A2402	NYLDLSALL	GH
120	CMV	A2402	QAIRETVEL	PP65
121	CMV	A2402	QYDPVAALF	PP65
122	CMV	A2402	QYDPVAALFF	PP65
123	EBV	A2402	DYNFVKQLF	ICP27
124	EBV	A2402	RYSIFFDYM	EBNA3
125	EBV	A2402	TYGPVFMCL	LMP2
126	EBV	A2402	PYLFWLAAI	LMP2
127	EBV	A2402	TYPVLEEMF	RTA
128	EBV	A2402	TYSAGIVQI	EBNA4
129	FLU-A	A2402	RYGFVANF	PB1
130	FLU-A	A2402	SWPDGAELPF	NA
131	HPV	A2402	QYNKPLCDL	VE6
132	SARS-COV	A2402	IYQTSNFRV	S
133	SARS-COV	A2402	NYMPYFFTL	ORF1
134	SARS-COV	A2402	NYNYLYRLF	S
135	SARS-COV	A2402	QYIKWPWYI	S
136	SARS-COV	A2402	SYATHSDKF	ORF1
137	SARS-COV	A2402	TYACWHHSI	ORF1
138	SARS-COV	A2402	VYFLQSINF	ORF3
139	SARS-COV	A2402	VYIGDPAQL	ORF1
140	CMV	A3002	KVRNIMKDK	IE2
141	CMV	A3002	RQYDPVAAL	pp65
142	FLU-A	A3002	YSHGTGTGY	PB1
143	FLU-A	A3101	ASCMGLIYNR	M1
144	CMV	A6801	ATTFLQTMLR	IE1
145	CMV	A6801	FVFPTKDVALR	PP65
146	CMV	A6801	TSAFVFPTK	PP65
147	HPV	A6801	LTAPTGCIKK	E2
148	CMV	A6801	YTPDSTPCHR	pp65
149	CMV	A6801	TTFLQTMLR	VIE1

150	EBV	A6801	ETFTETWNR	EAR
151	SARS-COV	A6801	STGSNVFQTR	Spike
152	CMV	B0702	RPHERNGFTVL	PP65
153	CMV	B0702	TPRVTGGGAM	PP65
154	EBV	B0702	IPMRPLRMQPI	EBNA4
155	EBV	B0702	LPWPTPKTHPV	EBNA6
156	EBV	B0702	QPLPGPQVTAV	EBNA4
157	EBV	B0702	QPRLTPPQPL	EBNA2
158	EBV	B0702	RPPIFIRRL	EBNA3
159	EBV	B0702	TPSVSSSISSL	BSRF1
160	EBV	B0702	WPMGYRTATL	EBNA6
161	EBV	B0702	YPRNPTEQGNI	MTP
162	HHV-1	B0702	VPRPDDPVL	TEG4
163	HRSV	B0702	NPKASLLSL	NCAP
164	JCPyV	B0702	IPVMRKAYL	F6LSF9
165	SARS-COV	B0702	APSASAFFGM	N
166	SARS-COV	B0702	FPRGQGVPI	N
167	SARS-COV	B0702	IPRRNVATL	ORF1
168	SARS-COV	B0702	KPRQKRTAT	N
169	SARS-COV	B0702	MPASWVMRI	ORF1
170	SARS-COV	B0702	SPRWYFYYL	N
171	SARS-COV	B0702	TPRDLGACI	ORF1
172	CMV	B0801	DANDIYRIF	PP65
173	CMV	B0801	DELRRKMMYM	VIE1
174	CMV	B0801	ELKRKMIYM	VIE1
175	CMV	B0801	ELKRKMMYM	VIE1
176	CMV	B0801	ELRRKMMYM	VIE1
177	CMV	B0801	QIKVRVDMV	VIE1
178	EBV	B0801	FLRGRAYGL	EBNA3
179	EBV	B0801	QAKWRLQTL	EBNA3
180	EBV	B0801	RAKFKQLL	BZLF1
181	EBV	B0801	RRRRRRAAL	EBNA6
182	EBV	B0801	YNLRRGIAL	EBNA1
183	FLU-A	B0801	ELRSRYWAI	NP
184	HEV	B0801	SALYRYNRF	ORF1_650
185	HHV-6B	B0801	DFKSKYLTL	IE2
186	SARS-COV	B0801	ITLKKRWQL	ORF3
187	SARS-COV	B0801	LPQGFSAL	S
188	SARS-COV	B0801	MIAQYTSAL	S
189	SARS-COV	B0801	QSASKIITL	ORF3
190	SARS-COV	B0801	TLDSKTQSL	S
191	EBV	B1501	GQGGSPTAM	EBNA4
192	EBV	B1501	LEKARGSTY	EBNA3
193	HEV	B1501	LMTYLRGISY	ORF1_340
194	SARS-COV	B1501	AQFAPSASAF	N
195	SARS-COV	B1501	HVGEIPVAY	ORF1
196	SARS-COV	B1501	KIEELFYSY	ORF1
197	SARS-COV	B1501	KMADQAMTQMY	ORF1
198	SARS-COV	B1501	LVKNKCVNF	S
199	SARS-COV	B1501	MSNLGMPSY	ORF1

200	SARS-COV	B1501	RVAGDSGFAAY	M
201	SARS-COV	B1501	VASQSIIAY	S
202	SARS-COV	B1501	YLITPVHVM	ORF1
203	EBV	B1801	SELEIKRY	BZLF1
204	FLU-A	B1801	YERMCNIL	NP
205	EBV	B2705	FRKAQIQGL	EBNA6
206	EBV	B2705	KRPPIFIRR	EBNA3
207	EBV	B2705	KRPPIFIRRL	EBNA3
208	EBV	B2705	RRRKGWIPL	BILF2
209	FLU-A	B2705	RRSGAAGAAVK	NP
210	HEV	B2705	GRYGRRTKL	ORF1_1315
211	HEV	B2705	RRRGFAAF	ORF1_1009
212	HEV	B2705	RRWLSAGFHL	ORF1_435
213	HEV	B2705	YRYNRFTQR	ORF1_653
214	SARS-COV	B2705	QRNAPRITF	N
215	CMV	B3501	HPTFTSQY	PP65
216	CMV	B3501	IPSINVHHY	PP65
217	EBV	B3501	EPLPQGQLTAY	BZLF1
218	EBV	B3501	HPVAEADYFEY	EBNA1
219	EBV	B3501	HPVGEADYFEY	EBNA1
220	EBV	B3501	HPVGQADYFEY	EBNA1
221	FLU-A	B3501	LPFERATIM	NP
222	FLU-A	B3501	LPFERATVM	NP
223	HEV	B3501	MAIIAHCY	ORF1_1535
224	HEV	B3501	WPADVAEAM	ORF1_170
225	HEV	B3501	YPLLGSGIY	ORF1_887
226	NWV	B3501	FPGEQLLFF	CAPSD
227	SARS-COV	B3501	IPFAMQMAY	S
228	SARS-COV	B3501	LPFNDGVYF	S
229	SARS-COV	B3501	QPTESIVRF	S
230	SARS-COV	B3501	TPSGTWLTY	N
231	SARS-COV	B3501	VPFWITIAY	ORF1
232	EBV	B3701	LDFVRFMGV	EBNA6
233	CMV	B4001	CEDVPSGKL	PP65
234	CMV	B4001	EEAIVAYTL	VIE1
235	CMV	B4001	HERNGFTVL	PP65
236	CMV	B4001	KEVNSQLSL	VIE1
237	EBV	B4001	IEDPPFNSL	LMP2
238	SARS-COV	B4001	GETLPTEVL	ORF1
239	SARS-COV	B4001	GEVITFDNL	ORF1
240	SARS-COV	B4001	GEAANFCAL	ORF1
241	SARS-COV	B4001	MEVTPSGTWL	N
242	SARS-COV	B4001	TEVVGDIIL	ORF1
243	HEV	B440X	MEECGMPQW	ORF1_1484
244	SARS-COV	B440X	EEAIRHVRAW	ORF1
245	SARS-COV	B440X	EEIAIILASF	ORF1
246	SARS-COV	B440X	MEVTPSGTW	N
247	SARS-COV	B440X	QEILGTVSW	ORF1
248	SARS-COV	B440X	QEYADVFHLY	ORF1
249	SARS-COV	B440X	SEFSSLPSY	ORF1

250	SARS-COV	B440X	YEQYIKWPW	S

Table S2. Virus peptide library with corresponding HLA restriction, sequence and peptide.

total n=22	bsl	ex02	ex60
CMV	5	5	5
EBV	21	22	20
Flu-A	1	1	1
HAdV-C	1	1	1
HEV	4	2	2
HHV-1	6	3	3
HHV-2	1	2	2
HHV-6B	13	15	13
HMPV	0	1	0
KSHV	4	7	4
NWV	0	0	1
SARS-CoV-2	21	22	20

Table S3. Number of patients with detectable peptide-specific CD8+ T cells grouped by associated virus.
